# The charge-transfer complex 1-amino­anthraquinone–7,7′,8,8′-tetra­cyano­quinodimethane (1/1)

**DOI:** 10.1107/S1600536813002195

**Published:** 2013-01-26

**Authors:** Adriano Bof de Oliveira, Johannes Beck, Jörg Daniels, Jaciara Nascimento Santos, Bárbara Regina Santos Feitosa

**Affiliations:** a Departamento de Química, Universidade Federal de Sergipe, Av. Marechal Rondon s/n, Campus, 49100-000 São Cristóvão-SE, Brazil; b Institut für Anorganische Chemie, Universität Bonn, Gerhard-Domagk-Strasse 1, D-53121 Bonn, Germany

## Abstract

The reaction of 1-amino­anthraquinone with 7,7′,8,8′-tetra­cyano­quinodimethane yielded the title charge-transfer complex, C_14_H_9_NO_2_·C_12_H_4_N_4_. The mol­ecules have maximum deviations from the mean planes through the non-H atoms of 0.0769 (14) Å for an oxo O atom and 0.1175 (17) Å for a cyano N atom, respectively. The dihedral angle between the two planes is 3.55 (3)°. In the crystal, mol­ecules are stacked into columns along the *a*-axis direction. Pairs of N—H⋯N and N—H⋯O inter­actions connect the mol­ecules perpendicular to the stacking direction. Additionally, an intra­molecular N—H⋯O hydrogen-bond inter­action is observed for 1-amino­anthraquinone.

## Related literature

For a revised structure of 1-amino­anthraquinone, see: Milić *et al.* (2012 ▶). For charge-transfer complexes of aromatic derivatives with 7,7′,8,8′-tetra­cyano­quinodimethane, see: Press *et al.* (2012 ▶). For the conductivity of organic salts, see: Jérome (2004 ▶). For the coordination chemistry of 7,7′,8,8′-tetra­cyano­quinodimethane, see: Kaim & Moscherosch (1994 ▶).
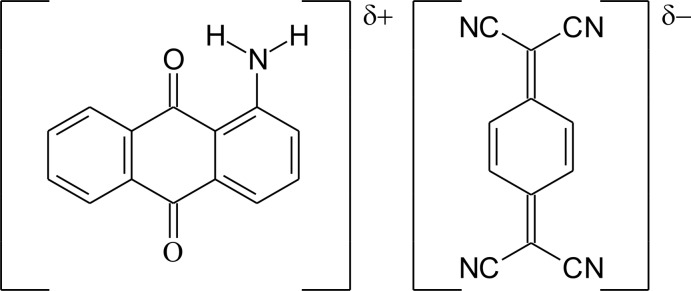


## Experimental

### 

#### Crystal data

C_12_H_4_N_4_·C_14_H_9_NO_2_
*M* *_r_* = 427.41
Monoclinic, 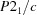

*a* = 7.4916 (2) Å
*b* = 9.4321 (3) Å
*c* = 28.8093 (8) Å
β = 95.8785 (15)°
*V* = 2025.00 (10) Å^3^
*Z* = 4
Mo *K*α radiation
μ = 0.09 mm^−1^
*T* = 293 K
0.29 × 0.05 × 0.04 mm

#### Data collection

Nonius KappaCCD diffractometer
Absorption correction: analytical (Alcock, 1970 ▶) *T*_min_ = 0.974, *T*_max_ = 0.996
19055 measured reflections
3972 independent reflections
2324 reflections with *I* > 2σ(*I*)
*R*_int_ = 0.147

#### Refinement

*R*[*F* ^2^ > 2σ(*F* ^2^)] = 0.052
*wR*(*F* ^2^) = 0.135
*S* = 1.01
3972 reflections
350 parameters
All H-atom parameters refined
Δρ_max_ = 0.16 e Å^−3^
Δρ_min_ = −0.16 e Å^−3^

### 

Data collection: *COLLECT* (Nonius, 1998 ▶); cell refinement: *SCALEPACK* (Otwinowski & Minor, 1997 ▶); data reduction: *DENZO* (Otwinowski & Minor, 1997 ▶) and *SCALEPACK*; program(s) used to solve structure: *SHELXS97* (Sheldrick, 2008 ▶); program(s) used to refine structure: *SHELXL97* (Sheldrick, 2008 ▶); molecular graphics: *DIAMOND* (Brandenburg, 2006 ▶); software used to prepare material for publication: *publCIF* (Westrip, 2010 ▶).

## Supplementary Material

Crystal structure: contains datablock(s) I, global. DOI: 10.1107/S1600536813002195/bt6884sup1.cif

Structure factors: contains datablock(s) I. DOI: 10.1107/S1600536813002195/bt6884Isup2.hkl

Supplementary material file. DOI: 10.1107/S1600536813002195/bt6884Isup3.cml

Additional supplementary materials:  crystallographic information; 3D view; checkCIF report

## Figures and Tables

**Table 1:** Hydrogen-bond geometry (Å, °)

*D*—H⋯*A*	*D*—H	H⋯*A*	*D*⋯*A*	*D*—H⋯*A*
N1—H*N*1⋯O2	0.93 (3)	1.96 (3)	2.654 (3)	130 (2)
N1—H*N*1⋯O2^i^	0.93 (3)	2.25 (3)	3.019 (3)	139 (2)
N1—H*N*2⋯N3^ii^	1.02 (3)	2.22 (3)	3.229 (3)	171 (2)

Symmetry codes: (i) 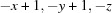; (ii) 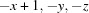.

**Fig. 1.**
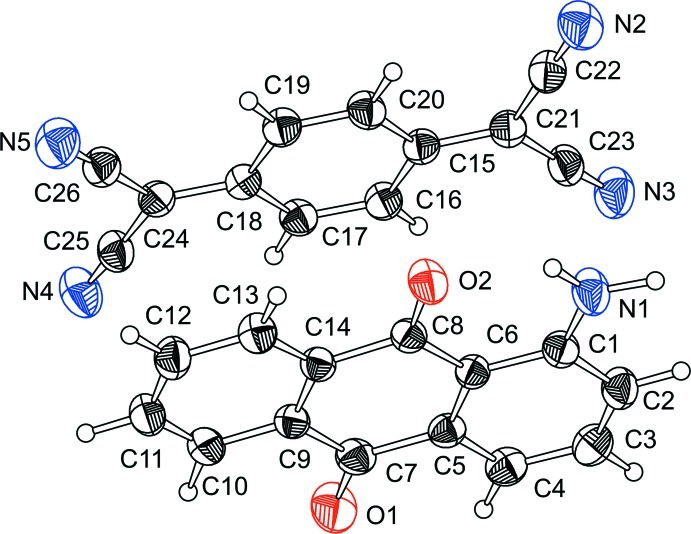
: The molecular structure of the title compound with labelling and displacement ellipsoids drawn at the 40% probability level.

**Fig. 2.**
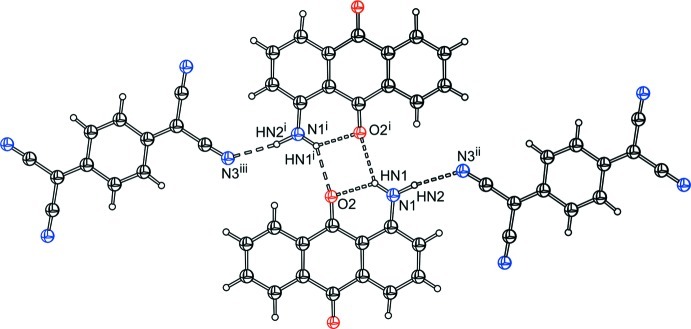
: Crystal structure of the title compound showing the dimeric arrangement. Intermolecular and Intramolecular hydrogen interactions are indicated as dashed lines. Symmetry codes: (i) -x + 1, -y + 1, -z; (ii) -x + 1, -y, -z; (iii) x, 1 + y, z.

**Fig. 3.**
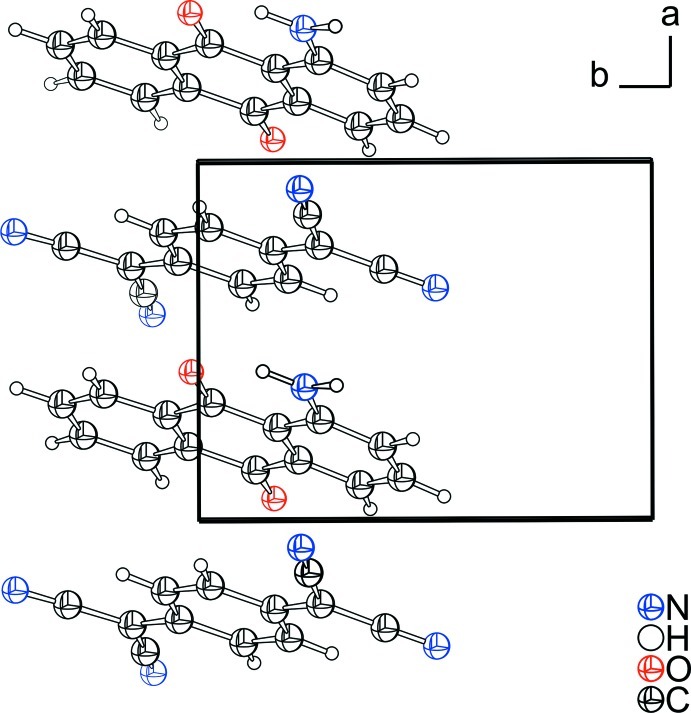
: Crystal structure of the title compound showing the mixed stacks of donor and acceptor molecules. The stacks run the crystallographic a axis. The graphical representation is simplified for clarity.

## supplementary crystallographic information

### Comment

Charge transfer compounds with 7,7',8,8'-tetracyanoquinodimethane (TCNQ) as the acceptor component have a wide range of properties, such as paramagnetism, cooperative magnetism or electrical conductivity. TCNQ can be easily reduced to the anionic form. The single unpaired electron occupies the lowest unoccupied molecular orbitals, that are mainly located at the terminal dicyanomethylene fragments. When the TCNQ units stack and the intermolecular spacings are shorter than the van der Waals distances for carbon, the π orbitals will contribute to electrical conductivity (Jérome, 2004). Additionally, TCNQ compounds have a very interesting coordination chemistry (Kaim & Moscherosch, 1994). As part of our study of charge transfer complex structures, we report herein the synthesis and the crystal structure of a new TCNQ-acceptor compound with 1-aminoanthraquinone-donor (Milić *et al.*, 2012).

In the title compound, the molecular structure unit matches the asymmetric unit (Fig. 1). Both molecules show only a slight deviation from planarity. The maximal deviation from the least squares plane through all non-hydrogen atoms for the 1-aminoanthraquinone and the 7,7',8,8'-tetracyanoquinodimethane molecule amount to 0.0769 (14) Å for O2 and 0.1175 (17) Å for N5, respectively, and the dihedral angle between the two planes is 3.55 (03)°. The bond angles suggest *sp*^2^ hybridization for the C atoms and explain the planarity of both molecules. The structure is built from mixed stacks of donor and acceptor molecules. The stacks run the crystallographic *a* axis (Fig. 3). The mean distance between the molecules within the stack amounts to one half of the length of the *a* axis, *i.e.* 3.7456 (2) Å. This explains the low electrical conductivity of the compound. Above room temperature, however, a detectable electrical conductivity was observed, which reaches 4.4 × 10^-8^ S/cm at 370 K. In the temperature range between 2 K and 300 K the title compound turned out as diamagnetic. The bond lengths within the dicyanomethylene groups suggest that the TCNQ units are neutral, comparing with crystal and infrared literature data (Kaim & Moscherosch, 1994). However, a small charge transfer is apparently present, since the electrical resistivity falls with increasing temperature indicating semiconducting characteristics. From the resistivity data, an Arrhenius development –ln(1/*R*) *versus*. 1/T gives a mainly linear behaviour, from which a small barrier for the thermally activated transport of 1.25 eV can be derived, according with dark brown colour of the crystals.

The crystal packing is stabilized by intermolecular hydrogen interactions. The molecules are connected by pairs of centrosymmetrical N—H···O and N—H···N hydrogen interactions, building dimers (Table 1; N1—HN1···O2^i^_;_ N1—HN2···N3^ii^ and Fig. 2). Additionally, an intramolecular N—H···O hydrogen interaction is observed for the 1-aminoanthraquinone (Table 1; N1—HN1···O2 and Fig. 2).

### Experimental

Starting materials were commercially available and were used without further purification. 1-Aminoanthraquinone and 7,7',8,8'-tetracyanoquinodimethane were dissolved in CH_2_Cl_2_ separately at room temperature and equimolar concentrations. The solutions were combined and maintained for 4 h under continuous stirring. Dark brown crystals, suitable for X-ray analysis, were obtained by the slow evaporation of the solvent. Elemental analysis: Calc. 73.1 C, 3.1 H, 16.4 N; found 72.8 C, 3.4 H, 16.6 N. The melting point was determined by differential scanning calorimetry to 520 K. Exothermic decomposition occurs at 555 K.

### Refinement

All hydrogen atoms were localized in a difference density Fourier map. Their positions and isotropic displacement parameters were refined.

### Crystal data

**Table:**

C_12_H_4_N_4_·C_14_H_9_NO_2_	*F*(000) = 880
*M**_r_* = 427.41	*D*_x_ = 1.402 Mg m^−^^3^
Monoclinic, *P*2_1_/*c*	Mo *K*α radiation, λ = 0.71073 Å
Hall symbol: -P 2ybc	Cell parameters from 14532 reflections
*a* = 7.4916 (2) Å	θ = 2.9–27.5°
*b* = 9.4321 (3) Å	µ = 0.09 mm^−^^1^
*c* = 28.8093 (8) Å	*T* = 293 K
β = 95.8785 (15)°	Needle, dark brown
*V* = 2025.00 (10) Å^3^	0.29 × 0.05 × 0.04 mm
*Z* = 4	

### Data collection

**Table:**

Nonius KappaCCD diffractometer	3972 independent reflections
Radiation source: fine-focus sealed tube	2324 reflections with *I* > 2σ(*I*)
Graphite monochromator	*R*_int_ = 0.147
Detector resolution: 9 pixels mm^-1^	θ_max_ = 26.0°, θ_min_ = 3.0°
CCD rotation images, thick slices scans	*h* = −9→9
Absorption correction: analytical (Alcock, 1970)	*k* = −11→11
*T*_min_ = 0.974, *T*_max_ = 0.996	*l* = −32→35
19055 measured reflections	

### Refinement

**Table:**

Refinement on *F*^2^	Primary atom site location: structure-invariant direct methods
Least-squares matrix: full	Secondary atom site location: difference Fourier map
*R*[*F*^2^ > 2σ(*F*^2^)] = 0.052	Hydrogen site location: inferred from neighbouring sites
*wR*(*F*^2^) = 0.135	All H-atom parameters refined
*S* = 1.01	*w* = 1/[σ^2^(*F*_o_^2^) + (0.0558*P*)^2^ + 0.1436*P*] where *P* = (*F*_o_^2^ + 2*F*_c_^2^)/3
3972 reflections	(Δ/σ)_max_ < 0.001
350 parameters	Δρ_max_ = 0.16 e Å^−^^3^
0 restraints	Δρ_min_ = −0.16 e Å^−^^3^

### Special details

**Table:**

Geometry. All e.s.d.'s (except the e.s.d. in the dihedral angle between two l.s. planes) are estimated using the full covariance matrix. The cell e.s.d.'s are taken into account individually in the estimation of e.s.d.'s in distances, angles and torsion angles; correlations between e.s.d.'s in cell parameters are only used when they are defined by crystal symmetry. An approximate (isotropic) treatment of cell e.s.d.'s is used for estimating e.s.d.'s involving l.s. planes.
Refinement. Refinement of *F*^2^ against ALL reflections. The weighted *R*-factor *wR* and goodness of fit *S* are based on *F*^2^, conventional *R*-factors *R* are based on *F*, with *F* set to zero for negative *F*^2^. The threshold expression of *F*^2^ > σ(*F*^2^) is used only for calculating *R*-factors(gt) *etc*. and is not relevant to the choice of reflections for refinement. *R*-factors based on *F*^2^ are statistically about twice as large as those based on *F*, and *R*- factors based on ALL data will be even larger.

### Fractional atomic coordinates and isotropic or equivalent isotropic displacement parameters (Å^2^)

**Table:**

	*x*	*y*	*z*	*U*_iso_*/*U*_eq_	
N1	0.6301 (3)	0.2662 (3)	0.00915 (7)	0.0621 (6)	
HN1	0.586 (3)	0.358 (3)	0.0048 (9)	0.086 (9)*	
HN2	0.625 (3)	0.195 (3)	−0.0176 (11)	0.110 (10)*	
N2	0.0775 (3)	0.2756 (2)	−0.02511 (7)	0.0705 (6)	
N3	0.3548 (3)	−0.0224 (2)	0.06905 (7)	0.0707 (6)	
N4	0.4265 (3)	0.6007 (2)	0.28057 (7)	0.0688 (6)	
N5	0.1954 (3)	0.9047 (2)	0.17933 (8)	0.0739 (6)	
O1	0.9414 (2)	0.33407 (19)	0.20941 (6)	0.0763 (5)	
O2	0.58619 (19)	0.51563 (15)	0.04905 (5)	0.0586 (4)	
C1	0.7206 (2)	0.2324 (2)	0.05065 (7)	0.0438 (5)	
C2	0.7953 (3)	0.0950 (2)	0.05632 (8)	0.0513 (6)	
H2	0.776 (3)	0.028 (2)	0.0292 (8)	0.070 (7)*	
C3	0.8834 (3)	0.0533 (3)	0.09792 (8)	0.0525 (6)	
H3	0.936 (3)	−0.040 (3)	0.1011 (8)	0.066 (7)*	
C4	0.9037 (3)	0.1446 (2)	0.13595 (8)	0.0485 (6)	
H4	0.971 (3)	0.122 (2)	0.1661 (9)	0.069 (7)*	
C5	0.8342 (2)	0.2799 (2)	0.13162 (7)	0.0407 (5)	
C6	0.7425 (2)	0.3273 (2)	0.08894 (7)	0.0376 (5)	
C7	0.8617 (2)	0.3753 (2)	0.17276 (7)	0.0473 (5)	
C8	0.6729 (2)	0.4722 (2)	0.08494 (7)	0.0397 (5)	
C9	0.7950 (2)	0.5229 (2)	0.16781 (7)	0.0413 (5)	
C10	0.8227 (3)	0.6157 (3)	0.20549 (8)	0.0524 (6)	
H10	0.895 (2)	0.5807 (18)	0.2367 (7)	0.039 (5)*	
C11	0.7634 (3)	0.7541 (3)	0.20053 (9)	0.0574 (6)	
H11	0.785 (3)	0.822 (2)	0.2272 (9)	0.072 (7)*	
C12	0.6755 (3)	0.8007 (3)	0.15876 (9)	0.0536 (6)	
H12	0.631 (3)	0.900 (2)	0.1552 (7)	0.061 (6)*	
C13	0.6477 (3)	0.7089 (2)	0.12139 (8)	0.0486 (5)	
H13	0.591 (3)	0.740 (2)	0.0917 (8)	0.052 (6)*	
C14	0.7067 (2)	0.5693 (2)	0.12546 (7)	0.0393 (5)	
C15	0.2500 (2)	0.3339 (2)	0.09187 (7)	0.0412 (5)	
C16	0.3302 (3)	0.3018 (2)	0.13811 (7)	0.0443 (5)	
C17	0.3431 (3)	0.4023 (2)	0.17152 (8)	0.0429 (5)	
C18	0.2788 (2)	0.5444 (2)	0.16208 (7)	0.0402 (5)	
C19	0.1987 (3)	0.5751 (2)	0.11561 (7)	0.0461 (5)	
C20	0.1842 (3)	0.4753 (2)	0.08236 (8)	0.0472 (5)	
C21	0.2352 (2)	0.2327 (2)	0.05729 (7)	0.0450 (5)	
C22	0.1486 (3)	0.2588 (2)	0.01153 (8)	0.0507 (6)	
C23	0.3026 (3)	0.0910 (3)	0.06488 (7)	0.0510 (6)	
C24	0.2928 (2)	0.6472 (2)	0.19600 (7)	0.0420 (5)	
C25	0.3679 (3)	0.6194 (2)	0.24281 (8)	0.0491 (5)	
C26	0.2367 (3)	0.7900 (3)	0.18669 (8)	0.0502 (5)	
H16	0.375 (2)	0.208 (2)	0.1455 (7)	0.053 (6)*	
H17	0.400 (3)	0.380 (2)	0.2023 (8)	0.055 (6)*	
H19	0.152 (3)	0.668 (2)	0.1072 (7)	0.059 (6)*	
H20	0.128 (3)	0.496 (2)	0.0507 (7)	0.054 (6)*	

### Atomic displacement parameters (Å^2^)

**Table:**

	*U*^11^	*U*^22^	*U*^33^	*U*^12^	*U*^13^	*U*^23^
N1	0.0828 (14)	0.0546 (14)	0.0455 (12)	0.0161 (11)	−0.0105 (10)	−0.0057 (11)
N2	0.0853 (14)	0.0770 (15)	0.0468 (12)	0.0103 (11)	−0.0045 (11)	0.0014 (11)
N3	0.0914 (14)	0.0511 (13)	0.0672 (14)	0.0174 (12)	−0.0032 (11)	−0.0074 (11)
N4	0.0893 (14)	0.0639 (14)	0.0503 (13)	−0.0007 (11)	−0.0069 (11)	−0.0034 (10)
N5	0.0883 (14)	0.0552 (14)	0.0758 (16)	0.0161 (12)	−0.0025 (12)	−0.0010 (12)
O1	0.0967 (12)	0.0819 (13)	0.0449 (10)	0.0278 (10)	−0.0190 (9)	0.0017 (9)
O2	0.0776 (10)	0.0491 (10)	0.0442 (9)	0.0158 (8)	−0.0171 (8)	0.0003 (7)
C1	0.0457 (11)	0.0459 (13)	0.0391 (12)	0.0034 (10)	0.0006 (9)	0.0000 (10)
C2	0.0595 (13)	0.0401 (14)	0.0534 (14)	0.0049 (10)	0.0016 (11)	−0.0044 (12)
C3	0.0567 (13)	0.0410 (14)	0.0598 (16)	0.0089 (11)	0.0060 (11)	0.0048 (12)
C4	0.0493 (12)	0.0493 (14)	0.0466 (13)	0.0059 (10)	0.0030 (10)	0.0084 (11)
C5	0.0384 (10)	0.0451 (13)	0.0383 (12)	0.0035 (9)	0.0029 (9)	0.0038 (10)
C6	0.0379 (10)	0.0387 (12)	0.0361 (11)	0.0022 (8)	0.0032 (9)	0.0009 (9)
C7	0.0444 (11)	0.0601 (15)	0.0363 (12)	0.0043 (10)	−0.0014 (9)	0.0034 (11)
C8	0.0396 (10)	0.0426 (12)	0.0363 (11)	0.0019 (9)	0.0006 (9)	0.0014 (10)
C9	0.0365 (10)	0.0484 (13)	0.0389 (12)	−0.0020 (9)	0.0040 (9)	−0.0044 (10)
C10	0.0470 (12)	0.0660 (17)	0.0432 (13)	0.0014 (11)	−0.0004 (10)	−0.0101 (12)
C11	0.0544 (13)	0.0640 (17)	0.0546 (15)	−0.0077 (12)	0.0082 (12)	−0.0193 (14)
C12	0.0574 (13)	0.0459 (15)	0.0588 (16)	−0.0040 (11)	0.0119 (12)	−0.0095 (12)
C13	0.0533 (12)	0.0455 (14)	0.0469 (14)	0.0005 (10)	0.0042 (11)	0.0012 (11)
C14	0.0372 (10)	0.0422 (13)	0.0386 (11)	−0.0023 (9)	0.0039 (9)	−0.0011 (9)
C15	0.0412 (10)	0.0406 (12)	0.0415 (12)	0.0033 (9)	0.0024 (9)	0.0020 (10)
C16	0.0485 (12)	0.0387 (13)	0.0447 (13)	0.0048 (10)	0.0000 (10)	0.0025 (11)
C17	0.0456 (11)	0.0413 (13)	0.0406 (12)	0.0030 (9)	−0.0011 (10)	0.0041 (11)
C18	0.0390 (10)	0.0396 (12)	0.0422 (12)	0.0006 (9)	0.0054 (9)	0.0006 (10)
C19	0.0511 (12)	0.0402 (13)	0.0460 (13)	0.0069 (10)	0.0005 (10)	0.0047 (11)
C20	0.0520 (12)	0.0484 (14)	0.0394 (12)	0.0052 (10)	−0.0047 (10)	0.0020 (11)
C21	0.0460 (11)	0.0444 (13)	0.0439 (12)	0.0036 (10)	0.0013 (9)	−0.0019 (10)
C22	0.0588 (13)	0.0481 (14)	0.0454 (14)	0.0050 (11)	0.0059 (11)	−0.0020 (11)
C23	0.0585 (13)	0.0515 (15)	0.0417 (12)	0.0050 (11)	−0.0016 (10)	−0.0072 (11)
C24	0.0418 (11)	0.0397 (12)	0.0441 (12)	0.0028 (9)	0.0021 (9)	0.0011 (10)
C25	0.0563 (13)	0.0400 (13)	0.0504 (14)	−0.0011 (10)	0.0028 (11)	−0.0045 (11)
C26	0.0533 (12)	0.0491 (15)	0.0473 (13)	0.0052 (11)	0.0013 (10)	−0.0023 (11)

### Geometric parameters (Å, º)

**Table:**

N1—C1	1.351 (3)	C10—C11	1.381 (3)
N1—HN1	0.93 (3)	C10—H10	1.055 (19)
N1—HN2	1.02 (3)	C11—C12	1.383 (3)
N2—C22	1.144 (3)	C11—H11	1.00 (2)
N3—C23	1.141 (3)	C12—C13	1.380 (3)
N4—C25	1.144 (3)	C12—H12	1.00 (2)
N5—C26	1.140 (3)	C13—C14	1.390 (3)
O1—C7	1.222 (2)	C13—H13	0.96 (2)
O2—C8	1.234 (2)	C15—C21	1.376 (3)
C1—C2	1.414 (3)	C15—C16	1.436 (3)
C1—C6	1.417 (3)	C15—C20	1.438 (3)
C2—C3	1.366 (3)	C16—C17	1.347 (3)
C2—H2	1.00 (2)	C16—H16	0.96 (2)
C3—C4	1.390 (3)	C17—C18	1.441 (3)
C3—H3	0.97 (2)	C17—H17	0.97 (2)
C4—C5	1.379 (3)	C18—C24	1.373 (3)
C4—H4	0.98 (2)	C18—C19	1.439 (3)
C5—C6	1.418 (3)	C19—C20	1.340 (3)
C5—C7	1.485 (3)	C19—H19	0.96 (2)
C6—C8	1.463 (3)	C20—H20	0.98 (2)
C7—C9	1.481 (3)	C21—C22	1.430 (3)
C8—O2	1.234 (2)	C21—C23	1.437 (3)
C8—C14	1.485 (3)	C24—C26	1.428 (3)
C9—C10	1.393 (3)	C24—C25	1.431 (3)
C9—C14	1.398 (3)		
			
C1—N1—HN1	118.7 (17)	C13—C12—C11	120.0 (2)
C1—N1—HN2	119.4 (17)	C13—C12—H12	119.2 (13)
HN1—N1—HN2	121 (2)	C11—C12—H12	120.8 (13)
N1—C1—C2	118.4 (2)	C12—C13—C14	120.5 (2)
N1—C1—C6	123.2 (2)	C12—C13—H13	121.5 (12)
C2—C1—C6	118.42 (19)	C14—C13—H13	118.0 (12)
C3—C2—C1	121.0 (2)	C13—C14—C9	119.24 (19)
C3—C2—H2	121.3 (13)	C13—C14—C8	119.39 (18)
C1—C2—H2	117.6 (13)	C9—C14—C8	121.37 (18)
C2—C3—C4	121.2 (2)	C21—C15—C16	121.35 (19)
C2—C3—H3	120.2 (13)	C21—C15—C20	120.32 (19)
C4—C3—H3	118.6 (13)	C16—C15—C20	118.33 (19)
C5—C4—C3	119.5 (2)	C17—C16—C15	120.5 (2)
C5—C4—H4	115.9 (13)	C17—C16—H16	119.3 (12)
C3—C4—H4	124.6 (13)	C15—C16—H16	120.2 (12)
C4—C5—C6	120.99 (19)	C16—C17—C18	121.4 (2)
C4—C5—C7	117.90 (18)	C16—C17—H17	119.6 (12)
C6—C5—C7	121.10 (18)	C18—C17—H17	118.9 (12)
C1—C6—C5	118.88 (18)	C24—C18—C19	120.93 (19)
C1—C6—C8	121.13 (17)	C24—C18—C17	121.53 (19)
C5—C6—C8	119.99 (18)	C19—C18—C17	117.54 (19)
O1—C7—C9	120.8 (2)	C20—C19—C18	121.3 (2)
O1—C7—C5	120.8 (2)	C20—C19—H19	117.4 (13)
C9—C7—C5	118.33 (17)	C18—C19—H19	121.4 (13)
O2—C8—C6	121.95 (18)	C19—C20—C15	121.0 (2)
O2—C8—C6	121.95 (18)	C19—C20—H20	121.3 (12)
O2—C8—C14	119.13 (18)	C15—C20—H20	117.7 (12)
O2—C8—C14	119.13 (18)	C15—C21—C22	122.92 (19)
C6—C8—C14	118.91 (17)	C15—C21—C23	122.28 (19)
C10—C9—C14	120.2 (2)	C22—C21—C23	114.79 (19)
C10—C9—C7	119.60 (19)	N2—C22—C21	178.0 (2)
C14—C9—C7	120.23 (18)	N3—C23—C21	177.3 (2)
C11—C10—C9	119.5 (2)	C18—C24—C26	122.18 (19)
C11—C10—H10	121.0 (10)	C18—C24—C25	122.36 (18)
C9—C10—H10	119.4 (10)	C26—C24—C25	115.45 (18)
C10—C11—C12	120.6 (2)	N4—C25—C24	178.2 (2)
C10—C11—H11	120.1 (13)	N5—C26—C24	178.6 (2)
C12—C11—H11	119.2 (13)		
			
N1—C1—C2—C3	−178.0 (2)	C10—C11—C12—C13	−0.6 (3)
C6—C1—C2—C3	1.3 (3)	C11—C12—C13—C14	0.3 (3)
C1—C2—C3—C4	−0.4 (3)	C12—C13—C14—C9	−0.2 (3)
C2—C3—C4—C5	−0.2 (3)	C12—C13—C14—C8	179.16 (18)
C3—C4—C5—C6	0.0 (3)	C10—C9—C14—C13	0.3 (3)
C3—C4—C5—C7	−178.83 (19)	C7—C9—C14—C13	−179.00 (17)
N1—C1—C6—C5	177.71 (19)	C10—C9—C14—C8	−179.07 (17)
C2—C1—C6—C5	−1.5 (3)	C7—C9—C14—C8	1.7 (3)
N1—C1—C6—C8	−2.6 (3)	O2—C8—C14—C13	−3.1 (3)
C2—C1—C6—C8	178.24 (17)	O2—C8—C14—C13	−3.1 (3)
C4—C5—C6—C1	0.9 (3)	C6—C8—C14—C13	177.34 (16)
C7—C5—C6—C1	179.67 (17)	O2—C8—C14—C9	176.20 (17)
C4—C5—C6—C8	−178.84 (17)	O2—C8—C14—C9	176.20 (17)
C7—C5—C6—C8	−0.1 (3)	C6—C8—C14—C9	−3.3 (3)
C4—C5—C7—O1	−0.9 (3)	C21—C15—C16—C17	−179.88 (19)
C6—C5—C7—O1	−179.75 (19)	C20—C15—C16—C17	−0.1 (3)
C4—C5—C7—C9	177.21 (16)	C15—C16—C17—C18	−0.4 (3)
C6—C5—C7—C9	−1.6 (3)	C16—C17—C18—C24	−179.60 (19)
O2—O2—C8—C6	0.00 (9)	C16—C17—C18—C19	0.4 (3)
O2—O2—C8—C14	0.00 (10)	C24—C18—C19—C20	−179.88 (19)
C1—C6—C8—O2	3.2 (3)	C17—C18—C19—C20	0.1 (3)
C5—C6—C8—O2	−177.04 (18)	C18—C19—C20—C15	−0.6 (3)
C1—C6—C8—O2	3.2 (3)	C21—C15—C20—C19	−179.6 (2)
C5—C6—C8—O2	−177.04 (18)	C16—C15—C20—C19	0.6 (3)
C1—C6—C8—C14	−177.26 (17)	C16—C15—C21—C22	176.89 (18)
C5—C6—C8—C14	2.5 (3)	C20—C15—C21—C22	−2.9 (3)
O1—C7—C9—C10	−0.3 (3)	C16—C15—C21—C23	−1.7 (3)
C5—C7—C9—C10	−178.49 (17)	C20—C15—C21—C23	178.58 (18)
O1—C7—C9—C14	178.94 (19)	C19—C18—C24—C26	−2.7 (3)
C5—C7—C9—C14	0.8 (3)	C17—C18—C24—C26	177.26 (18)
C14—C9—C10—C11	−0.5 (3)	C19—C18—C24—C25	178.53 (18)
C7—C9—C10—C11	178.78 (18)	C17—C18—C24—C25	−1.5 (3)
C9—C10—C11—C12	0.6 (3)		

### Hydrogen-bond geometry (Å, º)

**Table:**

*D*—H···*A*	*D*—H	H···*A*	*D*···*A*	*D*—H···*A*
N1—H*N*1···O2	0.93 (3)	1.96 (3)	2.654 (3)	130 (2)
N1—H*N*1···O2^i^	0.93 (3)	2.25 (3)	3.019 (3)	139 (2)
N1—H*N*2···N3^ii^	1.02 (3)	2.22 (3)	3.229 (3)	171 (2)

Symmetry codes: (i) −*x*+1, −*y*+1, −*z*; (ii) −*x*+1, −*y*, −*z*.
